# The decision to work after state pension age and how it affects quality of life: evidence from a 6-year English panel study

**DOI:** 10.1093/ageing/afx181

**Published:** 2018-01-10

**Authors:** Giorgio Di Gessa, Laurie Corna, Debora Price, Karen Glaser

**Affiliations:** 1 Institute of Gerontology, Department of Global Health and Social Medicine, King’s College London, London, UK; 2 School of Social Sciences, University of Manchester, Manchester, UK

**Keywords:** quality of life, CASP, English Longitudinal Study of Ageing, paid work, state pension age, older people

## Abstract

**Background:**

despite an increasing proportion of older people working beyond State Pension Age (SPA), little is known about neither the motivations for this decision nor whether, and to what extent, working beyond SPA affects quality of life (QoL).

**Methods:**

QoL was measured using the CASP-19 scale. Respondents in paid work beyond SPA were distinguished based on whether they reported financial constraints as the main reason for continuing in work. Linear regression models were used to assess the associations between paid work beyond SPA and CASP-19 scores among men aged 65–74 and women aged 60–69 (*n* = 2,502) cross-sectionally and over time using Wave 4 and Wave 7 of the English Longitudinal Study of Ageing.

**Results:**

approximately, one in five respondents were in paid work beyond SPA, one-third of whom reported financial issues as the main reason. These individuals reported significantly lower CASP-19 scores (*β* = −1.21) compared with those who retired at the expected/usual age. Respondents who declared being in paid work beyond SPA because they enjoyed their work or wanted to remain active, reported significantly higher QoL (*β* = 1.62). Longitudinal analyses suggest that those who were working post-SPA by choice, but who had stopped working at follow-up, also reported marginally (*P* < 0.10) higher CASP-19 scores.

**Conclusions:**

potential QoL benefits of working beyond SPA need to be considered in light of individual motivations for extending working life. Given the trend towards working longer and the abolishment of mandatory retirement ages, it is important that older people maintain control over their decision to work in later life.

## Introduction

In addressing the challenges posed by population ageing, governments continue to implement policy changes designed to extend working lives, including raising the state pension age (SPA) [1, 2], which has led to increasing ages at retirement [3]. Although labour market participation rates of older people are increasing [4], little is known about the reasons why some people work beyond SPA and whether, and to what extent, these affect their well-being.

Emphasis to date has largely been placed on understanding how retirees, rather than those still in work post-SPA, evaluate their post-retirement well-being by examining their reasons for retirement. Indeed, numerous studies have shown that having little or no control over the retirement transition has negative implications for quality of life (QoL) [5–7]. Yet, at a time when older adults are encouraged to work to ever-later ages, including beyond SPA, we know comparatively little about whether the motivations for this decision are also linked to QoL.

While continued work after SPA might provide workers with an opportunity to engage in physical, cognitive and social activities leading to higher QoL, a reverse effect could be hypothesised among those who feel that they ‘have to’ extend their working lives, particularly out of financial necessity. Thus, the two specific research questions we address in our study are as follows: why are older people in paid work beyond SPA? And are these reasons associated both cross-sectionally and longitudinally with QoL?

## Data and Methods

### Study population

We used data from the fourth and seventh waves of the English Longitudinal Study of Ageing (ELSA), collected in 2008/09 and 2014/15, respectively. ELSA is a multidisciplinary longitudinal survey representative of individuals aged 50 and over living in England (http://www.elsa-project.ac.uk/). Wave 4 of ELSA is the first wave to include questions about the reasons for working beyond SPA. We included respondents who had reached SPA (65 for men, 60 for women [8]) at this wave. We further restricted the sample to men aged 65–74 and women aged 60–69 as few men and women work beyond 74 and 69, respectively. We also excluded respondents who had never worked; who were ‘sick or disabled’ and who were ‘looking after home’ at baseline (as they were not asked the question about reasons for retirement); who died between waves; and with item missingness for the variables of interest. We based our analyses on 2,502 and 1,823 individuals for the cross-sectional and longitudinal analyses, respectively. Ethical approval for all the ELSA waves was granted by the Multi-centre Research and Ethics Committee (MREC).

### Main measurements of interest

*Subjective QoL*: This was evaluated using the CASP-19 scale, a validated measure specifically designed for individuals in later life used in a wide variety of ageing surveys [9–12]. CASP-19 is a 19-item self-completion questionnaire assessing four dimensions: control, autonomy, self-realisation and pleasure. The possible range of CASP-19 scores is from 0 to 57, with higher scores indicating greater well-being. CASP-19 was treated as a continuous variable. This variable is broadly normally distributed although slightly skewed left; in the study sample, the skewness of the baseline CASP-19 scale was −0.78 and its kurtosis was 3.62. Although CASP-19 was collected at all waves of ELSA, we used QoL measures three waves apart in line with previous studies as the time elapsed between each consecutive wave was too short for sufficient events and changes to have occurred in participants’ lives [13].

*Employment status and reasons*: We distinguished whether respondents were in paid work beyond SPA for financial reasons (i.e. because they ‘could not afford to retire earlier’ or wanted to ‘improve their pension/financial position’) or voluntarily (i.e. because they ‘enjoy working’ or to ‘keep active and fit’). Retirees were categorised into three main groups: ‘normal’ retirement (‘they reached SPA’); ‘involuntary’ retirement (due to their own or someone else’s ill health, or redundancy); and ‘voluntary’ retirement (including to ‘spend more time with family’ and because they ‘could afford to retire’).

When analysing the longitudinal associations between employment status and QoL, we considered stability and change between waves, distinguishing, for the latter, between those who were previously in the labour market for financial reasons and those who were working voluntarily post-SPA. As for the other transitions, only 19 retirees at Wave 4 were classified as in paid work at Wave 7. Also, among those in paid work at both waves it was not possible to study potential transitions between reasons for continuing to work beyond SPA as the questionnaire in Wave 7 no longer asked for the ‘main’ reason.

### Other covariates

In all multivariate analyses we adjusted for a wide range of potential confounders known to be associated with health and well-being [11, 13–17] as well as with the likelihood of working past SPA [18, 19]. Age was centred at SPA and a squared term was included as a covariate given that the relationship between QoL and age is non-linear [15]. Health measures included the presence of limiting long-standing illness (LSI); physical functioning (as the sum of any difficulties with 13 activities of daily living—ADL—and instrumental ADL); and depression (measured by an eight-item version of the validated CES Depression Scale [20]). As indicators of socio-economic circumstances, we included educational qualifications; quartiles of total net non-housing wealth; income; housing tenure (distinguishing outright owners, from those with a mortgage, and non-owners); and social class based on current or most recent job (distinguishing between managerial and professional; intermediate; and routine and manual occupations following the National Statistics Socio-Economic Classification). Indicators of social relationships included the respondent’s partnership status, and whether they reported volunteer work or informal care provision in the previous month. Three dimensions of social contacts were considered: the existence of close relationships (number of friends and family members with whom the respondent had close relationships); the presence of positive support (whether and to what extent relationships with friends and family members were based on understanding, support and confidence), and the frequency of contacts (how often family and friends were contacted or met). For all three dimensions we created summary scores, with high scores indicating greater quality and frequency of support.

Health and wealth indicators as well as social relationships and contacts were all considered both in terms of baseline levels and changes over the follow-up period. Changes were defined as difference in values between waves for summary variables (with positive values meaning improvements); and as ‘improvements’, ‘worsening’ or ‘no change’ (reference category) for categorical variables.

### Data analysis

Following descriptive findings to explore the baseline characteristics of the study population, we present cross-sectional linear regression models to determine whether QoL is associated with reasons for working beyond SPA. In a second step, we used conditional change multiple linear regression models to examine associations between changes in employment/retirement status and QoL at Wave 7, adjusting for CASP-19 score at Wave 4 [14], and for changes in the socio-economic and health covariates between waves. Under this approach, the regression coefficients indicate how the explanatory variables are associated with changes in QoL over time, since the initial score was controlled for [21]. Preliminary analyses were carried out separately for men and women but given similar findings, we present findings for the full sample only. All analyses were carried out using STATA SE version 14.1 [22].

## Results

### Descriptive findings

Almost 20% of the sample was in paid work beyond SPA. Of these, about two-thirds reported that they were in paid work because they ‘enjoy working’ or to ‘keep active and fit’, whereas the other third reported financial issues as the main reason for working beyond SPA. Among those ‘retired’ (80%), there was a similar distribution across categories of those reporting that they had retired voluntarily, involuntarily, or because they had reached SPA (in line with evidence from the early 2000s [23]). The CASP-19 score (range: 6–57, Wave 4 mean: 42.2) showed significant variation by employment status (see Table 1 for details). Respondents who were in paid work voluntarily reported the highest QoL (CASP-19 = 45.4) whereas the lowest QoL was reported by those who retired involuntarily (CASP-19 = 38.9). In order to more easily compare effect sizes, we found a similar difference of about 7 CASP-19 points between respondents who reported no LSI and those with a limiting LSI. As expected, respondents with more advantageous socio-economic characteristics and better health reported higher baseline CASP-19 scores. There is also a positive significant but weak correlation between social support and QoL (not shown).

**Table 1.** Baseline socio-economic, demographic and health characteristics of the study population, with unadjusted CASP-19 score in ELSA Wave 4

	*N*	%	W4 CASP-19 score [mean (SD)]	*P* value
Total sample	2,502	100	**42.2 (0.16)**	
Employment status				
Retired at SPA	696	27.8	41.9 (0.31)	<0.001
Voluntarily retired	694	27.7	44.4 (0.26)	
Involuntarily retired	616	24.6	38.9 (0.36)	
In paid work, financial necessity	169	6.8	41.0 (0.65)	
In paid work, voluntarily	327	13.1	45.4 (0.36)	
Health				
Without depressive symptoms	2,066	82.5	43.9 (0.15)	<0.001
With depressive symptoms	436	17.5	34.3 (0.43)	
No long-standing illness (LSI)	1,155	46.1	44.8 (0.21)	<0.001
With LSI	585	23.4	43.5 (0.28)	
With limiting LSI	762	30.5	37.5 (0.31)	
No physical limitation	1,988	79.5	43.8 (0.16)	<0.001
1 + Limitation (ADL/IADL)	514	20.5	36.5 (0.37)	
Socio-economic circumstances				
Some education	1,870	74.7	42.8 (0.18)	<0.001
No educational qualification	632	25.3	40.5 (0.34)	
In highest wealth quartile	613	24.6	45.0 (0.27)	<0.001
3rd Wealth quartile	632	25.2	43.7 (0.28)	
2nd Wealth quartile	632	25.2	41.7 (0.32)	
In lowest wealth quartile	625	25.0	37.8 (0.35)	
Own outright	1,853	74.1	43.2 (0.17)	<0.001
Own with mortgage	320	12.8	41.4 (0.50)	
Non-owners	329	13.1	37.5 (0.50)	
Managerial and professional	840	33.6	43.5 (0.26)	<0.001
Intermediate	664	26.5	42.5 (0.32)	
Routine and manual	998	39.9	40.9 (0.27)	
Social contact/relationship				
Volunteer	470	18.8	44.4 (0.31)	<0.001
Did not volunteer	2,032	81.2	41.7 (0.18)	
Cared for someone	336	13.4	41.4 (0.45)	0.054
Did not care for anyone	2,166	86.6	42.4 (0.17)	
Married/cohabiting	1,824	72.9	42.8 (0.18)	<0.001
Widowed	261	10.4	41.6 (0.48)	
Single/divorced/separated	417	16.7	40.0 (0.44)	
Demographic characteristics				
Men	1,091	43.6	41.9 (0.24)	0.016
Women	1,411	56.4	42.6 (0.22)	
Aged up to 5 years after SPA	1,391	55.6	42.6 (0.21)	0.023
Aged 6–10 years after SPA	1,111	44.4	41.9 (0.24)	

Source: ELSA Wave 4. Own calculations.

When we consider changes in CASP-19 scores over time (see Appendix A in the Supplementary data, available at Age and Ageing online), on average respondents experienced a decrease in QoL: about a quarter experienced a decrease of 5 points or more whereas just over 16% experienced an improvement of 5 or more points. Respondents who transitioned from employment ‘for financial reasons’ to retirement were the only subgroup who experienced an increase in their CASP-19 scores (on average, of almost 1 point) whereas no average changes were observed among those who retired but were previously working voluntarily (who still reported the highest CASP-19 score at Wave 7). Among retirees, the greatest reduction in QoL (and lowest CASP-19 score at Wave 7) was observed for those who indicated at baseline that they had retired involuntarily. Finally, as expected, QoL improved among respondents who experienced positive changes in their health status, and worsened for those whose health deteriorated.

### Multivariate analyses

To investigate whether the reasons for being in paid work beyond SPA were associated with QoL, we used multiple linear regression (Table 2). We present two nested models: the basic one which only adjusts for socio-economic and demographic characteristics, and the fully adjusted model which also accounts for health. This is because of variations in the health profile of respondents by reasons for retirement: those involuntary retired have the poorest health profile (27% were depressed; 38% had functional limitations; 52% reported limiting LSI) whereas those in work voluntarily tended to report better health (13% depressed; 15% with limiting LSI; 8% had functional limitation), with those retired at the normal age and in work for financial reasons somewhere in between.

**Table 2.** Basic and fully adjusted beta coefficients (with 95% CIs) and *P* values for the relationship between employment status beyond SPA and quality of life

	Model 1; Basic adjusted model		Model 2; Fully adjusted model	
	*B* (95% CI)	*P* value	*B* (95% CI)	*P* value
Retired at SPA	Ref		Ref	
Voluntarily retired	1.63 (0.84; 2.45)	<0.001	1.12 (0.42; 1.82)	<0.001
Involuntarily retired	−2.10 (−2.92; −1.28)	<0.001	−0.37 (−1.10; 0.36)	0.318
In paid work, financial necessity	−0.42 (−1.70; 0.86)	0.522	−1.21 (−2.34; −0.08)	0.035
In paid work, voluntarily	2.54 (1.52; 3.56)	<0.001	1.62 (0.69; 2.49)	<0.001
Age (years after SPA)	−0.08 (−0.53; 0.36)	0.698	−0.09 (−0.49; 0.30)	0.639
Age squared	−0.00 (−0.03; 0.04)	0.901	−0.00 (−0.04; 0.04)	0.992
Female (gender)	0.64 (0.01; 1.26)	0.045	1.21 (0.62; 1.71)	<0.001
No educational qualification^a^	−0.02 (−0.76; 0.71)	0.833	−0.10 (−0.75; 0.54)	0.756
3rd Wealth quartile^b^	−0.61 (−1.44; 0.22)	0.126	−0.55 (−1.28; 0.17)	0.136
2nd Wealth quartile^b^	−2.01 (−2.89; −1.13)	<0.001	−1.77 (−2.55; −1.00)	<0.001
In lowest wealth quartile^b^	−4.07 (−5.09; −3.06)	<0.001	−2.60 (−3.51; −1.70)	<0.001
Income (£10,000)	0.22 (−0.03; 0.44)	0.085	0.23 (0.03; 0.43)	0.026
Own with mortgage^c^	−1.08 (−2.02; −0.13)	0.026	−0.76 (−1.59; 0.07)	0.070
Non-owners^c^	−2.58 (−3.56; −1.61)	<0.001	−1.30 (−2.17; −0.44)	0.003
Intermediate^d^	−0.54 (−1.30; 0.23)	0.168	−0.51 (−1.18; 0.16)	0.138
Routine and manual^d^	−0.51 (−1.27; 0.26)	0.193	−0.43 (−1.10; 0.24)	0.211
Did not volunteer^e^	−1.25 (−2.03; −0.48)	<0.001	−0.64 (−1.32; 0.05)	0.067
Cared for someone^f^	−1.58 (−2.45; −0.72)	<0.001	−1.35 (−2.10; −0.59)	<0.001
Widowed^g^	−0.35 (−1.33; 0.63)	0.483	0.44 (−0.42; 1.31)	0.313
Single/divorced/separated^g^	−0.77 (−1.60; 0.06)	0.069	−0.51 (−1.24; 0.22)	0.169
Contacts with friends and family	0.14 (0.04; 0.24)	0.007	0.08 (−0.00; 0.17)	0.056
Quality of relationships	−0.02 (−0.05; 0.02)	0.334	−0.00 (−0.03; 0.03)	0.956
Number of close relationships	0.22 (0.16; 0.28)	<0.001	0.18 (0.13; 0.24)	<0.001
With depression^h^			−6.63 (−7.35; −5.91)	<0.001
With LSI^i^			−1.01 (−1.66; −0.36)	0.002
With limiting LSI^i^			−3.65 (−4.33; −2.97)	<0.001
Physical limitation			−0.89 (−1.13; −0.65)	<0.001
Constant	43.3 (41.4; 45.2)	<0.001	44.6 (42.7;46.0)	<0.001
Observations	2,502		2,502	
*R* squared	0.198		0.384	

Source: ELSA Wave 4. Reference categories:

^a^Some education

^b^in the highest wealth quartile

^c^own outright

^d^managerial and professional

^e^volunteered

^f^no care provided

^g^married or cohabiting

^h^no depression

^i^no long-standing illness (LSI). Own calculations.

In both models, the reasons for being in paid work were associated with CASP-19 scores. In Model 1, being in paid work out of financial necessity was not significantly associated with worse QoL compared to being retired at the expected/usual age; however, once health was adjusted for, this negative association became significant (*β* = −1.21). Conversely, respondents who reported being in paid work beyond SPA for positive reasons, reported significantly better QoL (*β*= 1.62). Among retirees, those who reported voluntary retirement were significantly more likely to report higher QoL (*β* = 1.12), whereas the association between reduced QoL and involuntary retirement observed in Model 1 (*β* = −2.10) is mostly accounted for by the poorer health profile of these respondents. As expected, better health and financial circumstances improved QoL scores as did number of close relationships and volunteering; however, caring for someone significantly reduced QoL.

Longitudinal multivariate models regressing QoL at Wave 7 on earlier QoL and change variables are presented in Table 3. Only respondents who stopped working between waves but who had previously worked beyond SPA voluntarily reported improvements in CASP-19 scores (*β* = 0.97), although once changes in health were accounted for this was only marginally significant (*P* < 0.10). In contrast, involuntary retirement 6 years earlier had an enduring negative effect on QoL (*β* = −1.59). As expected, improvements in the quality of relationships and in health were associated with higher CASP-19 scores at Wave 7. The negative effects of worsening health were more substantial for depression: becoming depressed was associated with an average decrease of almost 4 CASP units.

**Table 3.** Basic and fully adjusted beta coefficients (with 95% CIs) and *P* values for the conditional change model of CASP-19 score at Wave 7 compared with Wave 4

	Model 1; Basic adjusted model		Model 2; Fully adjusted model	
	*B* (95% CI)	*P* value	*B* (95% CI)	*P* value
Still retired at SPA	Ref		Ref	
Still voluntarily retired	−0.05 (−0.80; 0.71)	0.908	−0.09 (−0.84; 0.66)	0.817
Still involuntarily retired	−1.75 (−2.57; −0.92)	<0.001	−1.59 (−2.39; −0.79)	<0.001
Still In paid work	0.68 (−0.50; 1.87)	0.261	0.54 (−0.61; 1.69)	0.357
No longer in paid work, financial	1.03 (−0.08; 2.57)	0.187	0.58 (−0.91; 2.08)	0.442
No longer in paid work, voluntarily	1.15 (0.01; 2.29)	0.049	0.97 (−0.11; 2.08)	0.087
Age (years after SPA)	0.32 (−0.13; 0.76)	0.164	0.26 (−0.17; 0.69)	0.313
Age squared	−0.04 (−0.08; 0.00)	0.046	−0.04 (−0.07; 0.01)	0.079
Female (gender)	0.30 (−0.29; 0.90)	0.315	0.33 (−0.25; 0.91)	0.263
Wave 4 CASP score	0.72 (0.68; 0.76)	<0.001	0.70 (0.66; 0.74)	<0.001
Changes in income (£10,000)	0.07 (−0.16; 0.29)	0.552	0.11 (−0.33; 0.11)	0.337
Wealth^a^				
Improving	0.42 (−0.38; 1.22)	0.304	0.37 (−0.40; 1.15)	0.341
Worsening	−0.36 (−1.04; 0.31)	0.290	−0.26 (−0.91; 0.40)	0.442
Changes in relationships				
Contacts with friends and family	−0.09 (−0.18; −0.01)	0.037	−0.07 (−0.16; 0.02)	0.110
Quality of relationships	0.06 (0.03; 0.10)	<0.001	0.06 (0.02; 0.09)	<0.001
Number of close relationships	0.04 (−0.02; 0.10)	0.165	0.03 (−0.02; 0.09)	0.281
Depression^a^				
Improving			1.61 (0.60; 2.62)	0.002
Worsening			−3.83 (−4.86; −2.81)	<0.001
Long-standing illness^a^				
Improving			0.85 (0.06; 1.63)	0.034
Worsening			−0.63 (−1.31; 0.05)	0.047
Change in physical limitation			0.61 (0.36; 0.86)	<0.001
Constant	10.7 (8.63; 12.7)	<0.001	11.5 (9.42;13.6)	<0.001
Observations	1,823		1,823	
*R* squared	0.475		0.509	

Source: ELSA Waves 4 and 7. Study samples for changes in CASP-19 scores are restricted to those who responded to both interviews with no missing values (*N* = 1,823). Effects shown for wealth, social relationships and health are of the change in covariates over the follow-up period. *Note*: changes between waves in continuous variables are constructed such that positive values represent improvement.

^a^Reference category = ‘No change between waves’. Own calculations.

### Sensitivity analysis

We conducted sensitivity analyses to test the robustness of our models. In particular, we repeated our analyses (i) excluding the health question from CASP-19 in order to assess the impact of possible circularity; (ii) excluding depressed respondents at baseline in order to assess the potential overlap between clinical depression and QoL; (iii) using the Multiple Imputation approach (using chained equations for 20 cycles) which assumes that data are missing at random [24] in order to increase the statistical power of our analyses. The results obtained excluding the health question, and those for the imputed datasets were broadly similar to the ones presented above. However, when we excluded respondents who were depressed at baseline, involuntary retirement remained significantly associated with negative CASP-19 scores cross-sectionally, whereas those who stopped working following voluntary labour market participation reported improvements in QoL, even in the fully adjusted model. (Results of all analyses available from authors on request.)

## Discussion

In response to policy initiatives to extend working lives, an increasing percentage of people continue to work past SPA. We found that the motivations underpinning the decision to continue working past SPA exert an influence on QoL, similar to that found for retirement [6, 25, 26]. Those who continue to work for positive reasons (about two-thirds of workers) report the highest levels of QoL, similar to the levels reported by respondents with no long-standing illness. These workers also experience marginal improvements in QoL when they eventually leave the labour market, most likely because they have control over this transition. In contrast, those who continue working beyond SPA out of financial necessity (one third of workers) report a CASP-19 score of about 4 points lower at baseline, and this level does not rebound upon eventual retirement.

This study draws strength from focussing on a specific group of older people who have extended their working life beyond SPA. It also uses a valid and reliable measure of QoL for older adults [9–12], and carries out a number of sensitivity analyses to assess the robustness of our findings Our contribution, however, should be considered in light of several limitations. Because of the small numbers, we were not able to fully capture other key dimensions of work beyond SPA (e.g. working hours, physical demands of the job, whether respondents were self-employed or measures of occupational strain). Similarly, we were not able to consider those who returned to the labour market following an initial exit; and among those who retired over time, we could not further distinguish whether this was a voluntary or involuntary decision. Moreover, among those in paid work at both waves, it was not possible to explore whether the reasons for employment post-SPA changed over time as different information on this issue was collected in Wave 7. Finally, although our data come from a large nationally representative sample of the older population in England, it is worth noting that our study sample is skewed towards the more advantaged (as the percentage of those with no educational qualification is considerably lower than that reported in the census). It is therefore likely that our study underestimates the percentage of people who work out of financial necessity and the differences in QoL between those who retire or continue to work voluntarily and those constrained to continue work for financial reasons.

Our work contributes to an emerging body of evidence on inequalities in working longer and its association with health and well-being. Those with more disadvantaged socio-economic circumstances not only find themselves working longer than anticipated relative to their more advantaged counterparts [27], but the expected health benefits of longer working lives are not evident in empirical research. Indeed, working longer does not appear to confer health benefits [18, 19], and delaying retirement is linked with poorer health, such as greater cardiovascular disease—an effect that is especially pronounced among those with lower earnings and manual occupations [28]. We similarly find evidence of inequality with those who continue working beyond SPA out of economic necessity reporting lower QoL compared to those who work voluntarily (who report the highest levels of QoL), and this level is unlikely to rebound upon eventual retirement. In light of QoL’s association with adverse health outcomes, including mortality [29, 30], this is particularly worrisome.

Understanding why people continue working past SPA, and its implications for health and well-being across social groups is important given the potential for some social groups to be disproportionately disadvantaged by longer working lives. While initiatives aimed at helping workers maintain control over the decision to extend their employment are worthwhile, policy makers must also consider mechanisms to support individuals across the life course to ensure a minimum financial well-being in later life in order to mitigate the negative implications for QoL of having to work longer.

**Key points**

- No prior population-based study examined the link between reasons for working post state pension age (SPA) and quality of life.
- About one third of those in paid work past SPA reported financial-related issues as the main reason for this decision.
- Paid work beyond SPA out of economic necessity is associated with lower quality of life (measured with CASP-19 scores).
- Individuals with control over their decision to work past SPA report the highest quality of life.
- There is a lasting negative association between involuntary retirement and quality of life.

## Supplementary Material

Supplementary Data
