# Supplementary material for: The decision to work after state pension age and how it affects quality of life: evidence from a 6-year English panel study
Source: Age Ageing. 2018 Jan 10;47(3):450–7. doi: 10.1093/ageing/afx181 (PMC5920338; doi:10.1093/ageing/afx181)
Supplement: Supplementary Data [file reasonstoworkpostspaandqol-supplementarydata.docx]

**APPENDIX A. Description of changes in CASP score by changes in employment and in a selected number of characteristics between Wave 7 and Wave 4**

|  | **N** | **%** | **Change in CASP score**  **[Mean (SD)]** | **P value** | **CAPS at Wave 7** |
| --- | --- | --- | --- | --- | --- |
| **Employment status** |  |  |  |  |  |
| *Still Retired at SPA* | 538 | 29.5 | -0.71 (0.31) | <0.001 | 41.8 |
| *Still Voluntarily retired* | 543 | 29.8 | -1.24 (0.26) |  | 43.5 |
| *Still Involuntarily retired* | 428 | 23.5 | -1.70 (0.37) |  | 37.9 |
| *Still In paid work* | 54 | 3.0 | -0.38 (0.49) |  | 44.2 |
| *No longer in paid work, financial reason* | 87 | 4.7 | 0.92 (0.76) |  | 42.9 |
| *No longer in paid work, voluntarily* | 173 | 9.5 | -0.01 (0.40) |  | 45.2 |
|  |  |  |  |  |  |
| **Depression** |  |  |  |  |  |
| *No changes* | 1,502 | 82.4 | -0.96 (0.16) | <0.001 | 42.8 |
| *Improving* | 162 | 8.9 | 2.48 (0.60) |  | 40.6 |
| *Worsening* | 159 | 8.7 | -4.19 (0.53) |  | 35.4 |
| **Long-standing illness** |  |  |  |  |  |
| *No changes* | 1,101 | 60.4 | -0.83 (0.21) | 0.047 | 41.9 |
| *Improving* | 301 | 16.5 | 0.47 (0.35) |  | 42.6 |
| *Worsening* | 421 | 23.1 | -2.19 (0.31) |  | 41.6 |
| **Marital status** |  |  |  |  |  |
| *No changes* | 1,723 | 94.5 | -1.00 (0.15) | 0.173 | 42.0 |
| *No longer married* | 78 | 4.3 | 0.11 (1.07) |  | 41.9 |
| *Became married* | 22 | 1.2 | 0.69 (1.68) |  | 44.4 |
| **Wealth** |  |  |  |  |  |
| *No changes* | 986 | 54.1 | -0.99 (0.21) | 0.131 | 41.8 |
| *Improving* | 323 | 17.7 | -0.29 (0.38) |  | 42.1 |
| *Worsening* | 514 | 28.2 | -1.21 (0.29) |  | 42.4 |

Source: ELSA Wave 4 and Wave 7. Study samples for changes in CASP-19 scores are restricted to those who responded to both interviews (N=1,823).
